# The Prevalence of Oral Anomalies Among Healthy Newborns at a Gynecological Obstetric Hospital in Quito, Ecuador: An Observational, Cross-Sectional Study

**DOI:** 10.3390/dj13040158

**Published:** 2025-04-02

**Authors:** Eleonor Vélez-León, Emilia Guerrero, Mauricio Orlando Carrillo, Marina Cabrera, Gustavo Tello, Patricia Pinos

**Affiliations:** 1 Unidad Académica de Salud y Bienestar, Facultad de Odontología, Universidad Católica de Cuenca, Cuenca 010107, Ecuador; mvelezl@ucacue.edu.ec (E.V.-L.); ppinos@ucacue.edu.ec (P.P.); 2 Grupo de Investigación Innovación y Desarrollo Farmacéutico en Odontología, Facultad de Odontología, Jefatura de Investigación e Innovación, Universidad Católica de Cuenca, Cuenca 010107, Ecuador; 3 Latin American Network of Research on Fluorides and Dental Fluorosis, Cartagena 130009, Colombia; 4 Escuela de Odontología, Universidad Central del Ecuador, Quito 170527, Ecuador; carrillohinojoza1@hotmail.com (M.O.C.); macabrera@uce.edu.ec (M.C.); 5 Odontología Pediátrica, Universidad Nacional Federico Villarreal, Lima 15001, Peru; ptello@unfv.edu.pe

**Keywords:** newborns, neonates, oral manifestations, anomalies

## Abstract

**Objectives:** Early observations often fail to detect anomalies that may impact the health and quality of life of newborns. This study aimed to determine the prevalence of oral anomalies in newborns and explore their possible associations with sociodemographic factors. **Methods:** A cross-sectional study was conducted in Quito, Ecuador, analyzing a sample of 241 newborns. The presence of oral anomalies was recorded, and their association with sex, birth weight, maternal age, and gestational factors was evaluated. **Results:** The prevalence of oral anomalies was 72.3%, with Epstein’s pearls being the most common (50%). Other findings included Bohn’s nodules and dental lamina cysts, while no cases of natal teeth or congenital epulis were observed. Ankyloglossia was identified in 1.36% of newborns. No significant correlations were found between the presence of oral anomalies and sex, birth weight, maternal age, or gestational factors. **Conclusions:** The high prevalence (72.3%) of oral anomalies in the studied newborns underscores the importance of early detection and management. Epstein’s pearls were the most frequent anomaly, followed by Bohn’s nodules, dental lamina cysts, and ankyloglossia, while no cases of natal teeth or congenital epulis were identified. No statistically significant associations were found with sex, birth weight, maternal age, or pregnancy complications. These findings emphasize the need for early pediatric dental assessment and an interdisciplinary approach to ensure proper neonatal oral health. Further research is recommended to explore potential causes and interventions to optimize oral health from birth.

## 1. Introduction

The oral cavity of a newborn is unique and can exhibit various peculiarities, as well as alterations that can be defined as a set of anomalies affecting the jaws, palate, floor of the mouth, tongue, and lips. These disorders are often the result of altered development during embryogenesis or originate from events that occur in the uterus, impacting embryonic and fetal growth [1,2,3]. The oral cavity of newborns also hosts a dynamic microbiome influenced by various factors, including the mode of delivery, feeding practices, and early environmental exposure, which contribute to the early colonization and maturation of the oral ecosystem [4].

Oral mucosal lesions exhibit considerable epidemiological variability across different populations and geographic regions. Research in Argentina [5] observed a prevalence of 61.3% in pediatric patients aged 6 to 13 years, while a study in South Africa found an overall prevalence of 32.9% among over a thousand patients [6]. In Mexico, a lower prevalence of 7.4% was reported in a sample of children and adolescents aged 1 to 16 years [7]. These findings highlight the heterogeneity in the distribution of oral mucosal lesions, suggesting that genetic predisposition, environmental factors, and socioeconomic conditions contribute to their occurrence and variability across populations.

Among the most common findings are inclusion cysts, ankyloglossia, congenital epulis, and the presence of natal/neonatal teeth [8,9]. Although most of these alterations are generally asymptomatic, isolated, and tend to resolve over time without the need for treatment, some may be indicative of systemic diseases. This can cause concern for both neonatology professionals and parents [1,8,10].

Fromm [11] categorized inclusion cysts based on their location, distinguishing between Epstein’s pearls (EPs) located in the mid-palatal raphe or near the junction of the hard and soft palates, Bohn’s nodules (BNs) on the dental ridges, and dental lamina cysts (DLCs) on the alveolar crest [9,12,13].

BNs are cysts containing ductal and acinar cells, presenting as rounded, pearly white papules either individually or in clusters in approximately 47% of newborns [1,8]. Despite their high prevalence, they typically resolve between the second week and the fifth month after birth [8].

EPs are keratin cysts that appear as small, clustered, whitish papules. Their prevalence is around 35% in newborns, with no gender preference [8,14]. Some authors state that both EPs and BNs can be found in 65 to 85% of neonates [15]. With a lower prevalence, DLCs are found in the range of 25 to 53% of newborns [16]. These benign lesions originate from the dental lamina, are asymptomatic, and clinically present as multiple white or pink structures [8,17,18,19].

An anomaly related to the tongue of newborns has been identified, known as ankyloglossia (AG). Although there is still no consensus regarding its definition, this condition arises due to a short lingual frenulum that hinders the movement of the tip of the tongue [1,20]. The prevalence of AG ranges from 0.1% to 10.7%; this variation is, in part, attributable to differences among researchers. It is more common in infants (7%), with no significant difference between genders [1,8,10,21,22]. Its etiopathogenesis is not completely defined and may manifest either in isolation or in association with certain syndromes. This condition can be asymptomatic or have implications such as difficulties in breastfeeding, speech articulation problems, periodontal complications, malocclusion, and even psychological issues. In some cases, surgical interventions may be necessary to address this alteration [1,23,24].

Congenital epulis (CE), first described in 1871, is a benign tumor of granular cells that can occur in newborns. Clinically, it presents as a mass on the gum of varying sizes that emerges from the alveolar mucosa [25,26]. Although its etiology remains unknown, studies suggest a possible association with hormonal factors, as it is 8 to 10 times more common in females. Its incidence is 0.0006%, and it is three times more frequent in the maxilla than in the mandible [25,27]. This condition may manifest in a small form without symptoms, with the expectation of spontaneous resolution. However, in larger cases, it can lead to respiratory complications and swallowing difficulties. In such circumstances, early surgical removal becomes necessary [1,15,27] to ensure the well-being of the patient.

Teeth that appear at birth, known as natal teeth (NT), and those that erupt during the 30 days following birth, called neonatal teeth (NNT), are uncommon findings [28]. The prevalence is approximately 1 in 289 newborns for NT and 1 in 2212 for neonatal teeth [29]. Although the etiology of this condition is not fully defined, and its prevalence is not high, it causes concern among parents due to potential issues with feeding, discomfort during breastfeeding, and the possibility of developing ulcers near the teeth [28]. The treatment approach can vary from conservative options to surgical interventions [8,9,28,30].

Of the mentioned anomalies, AG, congenital epulis, and the presence of natal or NNT may adversely affect the quality of life of the newborn and could necessitate more invasive interventions [1,11,14,16,22,29]. It is crucial for healthcare professionals serving newborns to possess a comprehensive understanding of these anomalies. This knowledge enables timely detection and diagnosis, early treatment, prevention of related complications, and guidance for parents. Thus, the objective of this study was to determine the prevalence of oral alterations in newborns in Ecuador, contributing to closing the knowledge gap on the epidemiology of these anomalies in Latin America and allowing for comparisons with other regions of the world.

## 2. Materials and Methods

This is an observational, cross-sectional study on the prevalence of oral disorders in newborns at the Isidro Ayora Gynecological Obstetric Hospital (HGOIA) in Quito, Ecuador, in 2017. This research had the approval of the Research Committee of the HGOIA and the Ethics Committee of the Central University of Ecuador (approval code: UCEQ120417, approval date: 12 April 2017). The researchers obtained informed consent from the legal representatives of the participants.

### 2.1. Variables

This study assessed several dependent variables, with the primary focus on the overall prevalence of oral alterations in newborns. This primary dependent variable served as an indicator of the presence or absence of oral anomalies in the newborn population. Additionally, secondary dependent variables were analyzed, each representing the prevalence of specific types of alterations. These included Bohn’s nodules, Epstein’s pearls, dental lamina cysts, congenital epulis, natal or neonatal teeth, and ankyloglossia, thereby providing a comprehensive understanding of the spectrum of oral abnormalities in neonates. Moreover, this study examined numerous independent variables encompassing sociodemographic information. These included the neonate’s sex (male or female) and weight at birth (categorized as low, normal, or high), maternal age (classified into 14–18 years, 18–35 years, and over 36 years), gestational age (term, premature, or post-term), gestational history (primiparous or multiparous), pregnancy complications (including the absence or presence of urinary tract infection, anemia, diabetes, threatened miscarriage, toxoplasmosis, and asthma), and type of delivery (eutocic or dystocic). These variables were scrutinized to discern potential associations with the prevalence and types of oral alterations observed in newborns.

### 2.2. Sample

The sample size determination was obtained using proportion estimation for a finite population (N = 2100), with a confidence level of 95% and a precision of 5%, based on an expected proportion of 20%. This resulted in a sample size of 210 participants. Participants were assigned to this study through a simple random probability sampling method.

### 2.3. Criteria for Selection

Participants in this study were meticulously selected, following stringent criteria to establish a well-defined cohort. Inclusion criteria encompassed healthy newborns up to one week old and their mothers present in the postpartum wards, who provided informed consent for their infants to undergo intraoral clinical examination. Conversely, exclusion criteria comprised newborns subjected to prior interventions potentially impacting their oral cavity, such as oral surgeries or treatments. Additionally, infants with congenital abnormalities affecting the oral cavity were excluded to maintain sample integrity. Specific exclusion criteria included infants older than one week, those with craniofacial abnormalities at birth, newborns with systemic conditions necessitating intensive care, and infants diagnosed with syndromes at birth.

### 2.4. Calibration

The training and calibration in the process of assessing and diagnosing oral anomalies in newborns were conducted through an interobserver consensus process led by highly experienced professionals in the fields of pediatric dentistry and oral pathology. Two examiners, both experts in this area, participated in this critical adjustment phase. The training spanned three days, focusing on the accurate completion of the clinical record form and precise training to identify and differentiate the various types of pathologies under the following diagnostic criteria:

Oral mucosal cysts were identified by whitish changes with a macula or papule-like appearance, categorized according to Fromm’s classification [11]. This classification distinguishes between Epstein’s pearls (EPs) located in the mid-palatal raphe or near the junction of the hard and soft palates, Bohn’s nodules (BNs) on the dental ridges, and dental lamina cysts (DLCs) on the alveolar crest;

Ankyloglossia was diagnosed when the lingual frenum was closely attached to the border of the papillated part of the tongue, restricting tongue protrusion. Parameters were determined following the method of Martinelli [31];

Natal or neonatal teeth referred to the appearance of a tooth’s incisal edge breaking through the mucous membrane;

Congenital epulis was identified as a pedunculated soft swelling ranging from one millimeter to several centimeters in diameter, typically located in the anterior maxillary ridge and present at birth.

To ensure interobserver reliability beyond the kappa coefficient, a comprehensive validation process was conducted. Initially, a standardization and pilot phase was carried out, where a set of 50 neonatal oral photographs representing various anomalies was selected. Each examiner independently classified these images based on the predefined diagnostic criteria, and any discrepancies were discussed until a consensus was reached. Following this, live clinical calibration was performed, during which the two examiners independently assessed 20 newborns at different times within a controlled environment. Their findings were compared, and any inconsistencies were reviewed together to refine the diagnostic accuracy. To further validate the consistency, a repeated measures evaluation was conducted one week later with the same cases to assess the intra-examiner reliability. The results demonstrated a weighted kappa coefficient of 0.8, indicating substantial agreement, while additional calculations of percentage agreement revealed a 92% consistency rate in anomaly identification. Furthermore, the time allocated for each phase—both for explaining the procedure and conducting the clinical examination—was systematically recorded, averaging 10 min per session. This structured approach not only reinforced the statistical reliability but also ensured practical consistency in the diagnostic application, enhancing the study’s methodological robustness.

### 2.5. Examination

Before conducting the oral examination of newborns, relevant information about both the mother and the neonate was gathered. This included the mother’s medical record number and age, as well as the neonate’s identifying code, all noted to maintain anonymity. Maternal–neonatal data recorded encompassed the mother’s age at delivery, gestational age in weeks, pertinent gestational history, any pregnancy-related issues encountered, type of delivery, and clinical details of the newborn, such as weight, sex, and current oral conditions.

To facilitate the observation of newborns at the hospital, a series of protocols and vital preparations were undertaken. Sterile dental instruments were readied, and the availability of essential materials like the headlamp, mask, cap, and disposable gown was ensured. This observation was carried out in postpartum wards 1 and 2, after confirming that the medical records met the study’s selection criteria.

In the postpartum wards, following a comprehensive briefing on the procedure, including the completion of medical records and obtaining informed consent, the oral examination commenced. For newborn clinical examinations, mothers were asked to hold their babies in a supine position, either in their arms or on their abdomen. In cases where the mother had experienced a dystocic delivery, the infant was positioned on the bed next to her to facilitate observation. Subsequently, the newborn’s mouth was gently cleaned using sterile gauze and saline solution.

The clinical examination was conducted with sterile latex gloves using the index finger, following the assessment criteria outlined by the World Health Organization [32], and applying the aforementioned diagnostic criteria.

Upon the completion of each patient’s observation, used gloves, masks, disposable gowns, and shoe covers were properly disposed of in accordance with the Regulation for the Management of Infectious Waste for the Health Services Network in Ecuador, ensuring compliance with hygiene regulations and the prevention of contagion among patients.

### 2.6. Statistical Analysis

The analysis was initiated by examining the prevalence of oral anomalies in newborns, alongside the general characteristics of the parents and neonates, with the results presented as percentage frequencies. To investigate potential links between oral anomalies and sociodemographic factors, the chi-square test was utilized. Statistical analyses were conducted using IBM^®^ SPSS v.21 (New York, NY, USA) and Microsoft Excel (version 2021), with the significance level set at 5% (*p* < 0.05) [33].

Two hypotheses were formulated: (1) Null hypothesis (H0): There is no significant association between the presence of oral anomalies in newborns and the sociodemographic characteristics of parents and neonates. Specifically, factors such as the neonate’s sex and birth weight, mother’s age, and pregnancy complications are not correlated with the prevalence of oral anomalies. (2) Alternative hypothesis (H1): there is a significant association between the presence of oral anomalies in newborns and the specific sociodemographic characteristics of parents and neonates.

## 3. Results

### 3.1. General Prevalence and Distribution of Oral Anomalies in Neonates

The prevalence of oral alterations in newborns was 72.3% (*p* = 0.01), indicating a significant occurrence in the studied population. Among the most frequently observed anomalies, Epstein’s pearls had an overall prevalence of 49.6%, with 42.5% located along the midline (*p* = 0.01) and 7.1% occurring off the midline (*p* = 0.04). Similarly, the total prevalence of Bohn’s nodules was 34.0% (*p* = 0.02), with 30.6% located in the maxilla (*p* = 0.02) and 3.4% in the mandible (*p* = 0.05). Additional findings included dental lamina cysts, with prevalences of 15.7% in the maxilla (*p* = 0.02) and 5.3% in the mandible (*p* = 0.03), as well as ankyloglossia, which was observed in 2.5% of newborns (*p* = 0.04). Finally, the presence of natal teeth was identified in 1.2% of cases in the mandible (*p* = 0.05) and 1.8% in the maxilla (*p* = 0.05) (Table 1).

### 3.2. Distribution of Sociodemographic Characteristics of Newborns and Their Mothers

The data obtained indicate a balanced distribution of newborn sex, with a slight male predominance (55.91%) over female (44.09%), with statistical significance at *p* = 0.05. Regarding birth weight, the majority of neonates had a normal birth weight (97.27%, *p* = 0.01), while 2.27% were classified as low birth weight (*p* = 0.02) and only 0.45% were categorized as high birth weight (*p* = 0.03). With respect to maternal age, 70.91% of mothers were within the 18–35-year range (*p* = 0.04), whereas 20.45% were between 14 and 18 years (*p* = 0.05), and 8.64% were older than 36 years (*p* = 0.06). In terms of gestational age, the majority of deliveries were full-term (93.18%, *p* = 0.01), while 5.45% were preterm (*p* = 0.02) and 1.36% were post-term (*p* = 0.03).

Regarding parity, a nearly equal distribution was observed between primiparous (51.82%, *p* = 0.05) and multiparous (48.18%, *p* = 0.05) women. The absence of obstetric complications was reported in 70.45% of cases (*p* = 0.01), while the most frequently observed complications included urinary tract infections (20.91%, *p* = 0.02), anemia (3.64%, *p* = 0.03), gestational diabetes (2.27%, *p* = 0.04), and other complications (2.73%, *p* = 0.05). Finally, an equitable distribution was noted between delivery types, with 49.55% of births classified as eutocic (*p* = 0.02) and 50.45% as dystocic (*p* = 0.01). The aforementioned is visualized in Table 2.

### 3.3. Association Between Oral Alterations in Newborns and Maternal–Neonatal Variables

The data indicate no statistically significant association (*p* = 0.2) between premature infants and the presence of oral anomalies. Maternal age (18 to 35 years) does not show a statistically significant association with oral alterations in neonates (*p* = 0.81). Similarly, there is no significant association between maternal parity (primiparous vs. multiparous) and the presence of oral issues in newborns (*p* = 0.43) (Table 3).

Maternal health complications during pregnancy do not demonstrate a statistically significant correlation with a higher frequency of oral alterations in newborns (*p* = 0.83). Additionally, there is no significant impact of newborns’ sex and birth weight on the prevalence of oral alterations (*p* = 0.21 for sex and *p* = 0.16 for weight) (Table 3).

Thus, the null hypothesis (H0) is confirmed, indicating no significant association between the presence of oral anomalies in neonates and the sociodemographic characteristics of the parents and neonates.

## 4. Discussion

Oral disorders in newborns raise relevant considerations in public health and neo-natal medical care in Ecuador. These alterations can affect the sucking and feeding ability of the child, as well as generate delays in the development of speech and dental health in the short and long terms [32]. It is imperative to investigate in depth the factors that trigger these pathologies, among which exposure to environmental factors, maternal habits during pregnancy, the age of the mother, and genetic background, among others, have been reported; their identification could help to reduce these diseases and thus promote better oral health from an early age [1,9].

Research on oral alterations in newborns in Quito, Ecuador, sheds light on a common yet underestimated issue in child health. By closely examining the prevalence of these anomalies in 250 babies, it is revealed that approximately 72% exhibited at least one of these oral peculiarities, similar to another study conducted in Mexico, where 91.2% of neonates showed some anomalies in the oral cavity [9]. A similar study conducted in Brazil [8] showed a prevalence of 56.7% in a sample of 411 newborns, indicating that Ecuador significantly and alarmingly surpasses this rate. Data reported from Oviedo, Spain, correspond to 51.9% of the population affected with oral alterations, while in Turkey, the prevalence is 41.7% [9].

The cause of this discrepancy remains unclear, but it may be influenced by geographical variations, sociodemographic factors, and differences in diagnostic criteria and patient care protocols [13,33]. Additionally, racial and ethnic factors have been associated with variations in oral cyst prevalence, with studies suggesting a higher occurrence among Caucasian newborns compared to African-American newborns [8]. However, since all neonates in our study were of Ecuadorian mestizo ethnicity, racial or ethnic comparisons were not possible.

A key finding of this study is the lack of a significant association between oral anomalies and sociodemographic factors such as maternal age, birth weight, and pregnancy complications. This contrasts with previous research, such as that by Pérez et al. [9], who reported a significant correlation between maternal folic acid intake and neonatal oral cysts. Although all participants in our study reported prenatal vitamin supplementation, the adherence could not be objectively verified due to the cross-sectional study design. Moreover, critical variables, including the prenatal care quality, maternal nutrition, and environmental exposures, were not comprehensively evaluated, which may have influenced the results.

Methodological and population characteristics likely contributed to the observed lack of association. The homogeneity of the study population in terms of ethnicity and socioeconomic background may have limited the detection of genetic or lifestyle-related influences on neonatal oral anomalies. Additionally, discrepancies between studies may be due to differences in their diagnostic criteria and research methodologies. For instance, studies in Brazil [8] and Mexico [9] identified significant associations between oral anomalies and factors such as preterm birth, maternal employment, and neonatal weight, which were not observed in our study. These inconsistencies highlight the need for standardized diagnostic protocols and multi-center research to better understand the complex interplay of factors influencing neonatal oral health.

Regarding the specific anomalies observed, Epstein’s pearls (EPs), Bohn’s nodules (BNs), and dental lamina cysts were the most prevalent, aligning with previous literature [9,13,15]. These lesions, despite their distinct etiologies, have been historically misclassified due to their clinical, histological, and evolutionary similarities. In our study, BNs were identified in 34% of neonates, without significant associations with any of the analyzed variables. This contrasts with the findings of Pérez et al. [9], who reported a statistically significant relationship between maternal folic acid intake and the presence of these lesions. However, this association remains uncertain, as compliance with prenatal supplementation could not be objectively verified due to the study’s design. Further research is needed to explore the potential link between folic acid intake and oral inclusion cysts or other neonatal oral anomalies.

In this particular study, EPs were observed to be the most prevalent anomaly, with almost 50% of cases presenting these pearls. Furthermore, 42,5% of these pearls were located specifically in the midline of the hard palate in the maxilla. These findings are consistent with previous research that also identified EPs as the most frequent anomaly, with 38.3% of cases located in the midline [8]. However, it is important to note that there are discrepancies among different studies, as highlighted in the research conducted in Mexico [9], where an even higher prevalence of 66% was reported. These variations may stem from differences in the studied population, evaluation methods, or even environmental factors. In a Brazilian study conducted in 2022, EPs were found to have a prevalence of 39.9%, making them the most commonly observed anomaly. This prevalence was associated with factors such as prematurity (*p* = 0.025), low birth weight (*p* = 0.033) in newborns, and maternal employment during pregnancy (*p* = 0.019) [10].

Some prior studies suggest that gestational age may inversely impact the prevalence of EPs [34,35]. Our findings align with those of another study [9], where no association was found between gestational age and birth weight with the occurrence of EPs.

AG, linked to difficulties with latch and maternal pain, identified as two of the main reasons behind breastfeeding cessation [36], can be responsible for complications in infant feeding [20,36]. This study reports a prevalence of 2.5% of this condition, with no significant differences found regarding the analyzed variables. The presence of the frenulum is observed mainly in patients without pathologies or congenital diseases [9,20,37], and there is even evidence suggesting a possible genetic transmission of this peculiarity [37]. Additionally, an association has been reported between newborns of mothers who consumed cocaine during pregnancy and AG [20,38].

The absence of standardized, objective clinical criteria for the diagnosis of AG has led to variability in the prevalence rates reported in the literature, ranging from 4% to 10% [20,38,39,40]. The diversity in the methodological approaches used in its study makes it difficult or even impossible to compare between studies.

In this study, neonatal teeth (NNT) were found in less than 2% of the studied population, with a higher prevalence in the mandible. Globally, approximately 1 in 289 newborns presents with natal teeth, and 1 in 2212 presents with neonatal teeth [30]. Although this prevalence is not high, it is crucial for professionals to be vigilant about identifying these conditions, as they often require immediate attention.

One of the main factors affecting the prevalence of these teeth in newborns is genetics. It has been observed that inheritance plays a significant role in the presence of neonatal teeth, with some studies suggesting that certain populations may have a higher genetic predisposition to this phenomenon [28].

In addition to genetics, other factors that may influence the prevalence of NNT include the presence of certain underlying medical conditions in the mother during pregnancy, such as Down syndrome, and certain environmental factors. It has been suggested that exposure to environmental toxins or certain medications during pregnancy may increase the likelihood of a newborn developing neonatal teeth [28,30]. Other factors that may play a role in the prevalence of NNT include maternal age, nutrition during pregnancy, and maternal dental health. Further research is needed to fully understand all the factors influencing the prevalence of NNT and to develop appropriate prevention and management strategies for this phenomenon.

Interestingly, no NT or CE were found, providing a clearer picture of the observed findings. The lack of significant associations between these anomalies and factors such as the baby’s sex and birth weight, maternal age, or pregnancy complications raises questions about the origin and underlying causes of these conditions. The contrast in the prevalence of these anomalies in Ecuador compared to that in other countries suggests the need for additional research to better understand this phenomenon and establish effective preventive strategies.

This study has several limitations that may affect the generalizability and validity of its findings. First, the sample size of 241 newborns may not fully represent the neonatal population of Quito, Ecuador, limiting the extrapolation of the results to a broader context. Additionally, the cross-sectional design precludes the establishment of causal relationships between the identified oral anomalies and the analyzed sociodemographic factors. The potential for selection bias in participant recruitment could also have influenced the reported prevalence rates, as the reliance on clinical examinations and medical records may have introduced variability into the data accuracy and completeness.

Another limitation is the lack of long-term follow-up, which restricts the ability to assess the impact of neonatal oral anomalies on oral health and overall development during childhood. Additionally, the limited statistical power of this study may reduce the likelihood of detecting significant associations between oral anomalies and sociodemographic variables.

Furthermore, the data were collected in 2017, which raises concerns regarding the current relevance of the findings. Over the past seven years, advancements in neonatal care, prenatal nutrition, and diagnostic criteria may have influenced the prevalence and recognition of these conditions. Although this study provides valuable epidemiological insights, its findings should be interpreted within the context of potential changes in clinical practices and public health policies. Future research should reassess these anomalies in a contemporary framework to determine whether the observed trends persist. Lastly, unexamined external factors, including environmental influences, maternal habits such as smoking, and genetic predispositions, may contribute to the development of neonatal oral anomalies. Addressing these limitations in future research is essential for enhancing the understanding of neonatal oral health and developing effective early detection and intervention strategies.

These findings underscore the need for interdisciplinary health policies that integrate neonatal oral health into comprehensive medical care, highlighting the importance of a collaborative approach to achieve optimal health outcomes

## 5. Conclusions

This research reveals a high prevalence (72.3%) of oral abnormalities among the newborns analyzed at the HGOIA in Quito, Ecuador. Epstein’s pearls emerged as the most common anomaly observed in these neonates, followed by Bohn’s nodules, dental lamina cysts, and ankyloglossia. No cases of natal teeth or congenital epulis were detected in the studied sample. Although oral anomalies were frequent, statistically significant associations were not established with variables such as sex, birth weight, maternal age, or pregnancy complications. These findings underscore the importance of the early detection and proper management of oral anomalies in newborns to promote oral health from an early age. The need to investigate the causes and possible interventions for these anomalies, as well as the importance of early pediatric dental assessment and an interdisciplinary approach to their management, are emphasized. Together, these results highlight the importance of addressing oral anomalies in newborns to promote their oral health and overall well-being from the earliest stages of life.

## Figures and Tables

**Table 1:** Distribution of oral alterations of newborns.

Oral Alteration	Prevalence (%)	*p*-Value
Ankyloglossia—presence	2.5	0.04
Neonatal teeth—mandible	1.2	0.05
Neonatal teeth—maxilla	1.8	0.05
Dental lamina cysts—mandible	5.3	0.03
Dental lamina cysts—maxilla	15.7	0.02
Epstein’s pearls—off midline	7.1	0.04
Epstein’s pearls—midline	42.5	0.01
Bohn’s nodules—presence in the mandible	3.4	0.05
Bohn’s nodules—presence in the maxilla	30.6	0.02
Oral alterations—yes	72.3	0.01

Note: Prevalence (%): percentage of newborns affected by each alteration. *p*-Value: statistical significance level (*p* < 0.05 indicates significant difference).

**Table 2:** Distribution of sociodemographic characteristics of newborns and their mothers.

Characteristic	Percentage (%)	*p*-Value
Newborn Gender—Male	55.91	0.05
Newborn Gender—Female	44.09	0.05
Birth Weight—Normal	97.27	0.01
Birth Weight—Low	2.27	0.02
Birth Weight—High	0.45	0.03
Maternal Age 18–35 years	70.91	0.04
Maternal Age 14–18 years	20.45	0.05
Maternal Age > 36 years	8.64	0.06
Gestational Age—Term	93.18	0.01
Gestational Age—Preterm	5.45	0.02
Gestational Age—Post-term	1.36	0.03
Parity—Primiparous	51.82	0.05
Parity—Multiparous	48.18	0.05
No Pregnancy Complications	70.45	0.01
Complication—UTI	20.91	0.02
Complication—Anemia	3.64	0.03
Complication—Diabetes	2.27	0.04
Other Complications	2.73	0.05
Delivery—Eutocic	49.55	0.02
Delivery—Dystocic	50.45	0.01

Note: Prevalence (%): percentage of newborns affected by each characteristic. *p*-Value: statistical significance level (*p* < 0.05 indicates a significant difference).

**Table 3:** Association between oral alterations in newborns and maternal–neonatal variables.

Category	Percentage (%)	*p*-Value
Weight of the newborn—normal weight	0.46	0.16
Weight of the newborn—low birth weight	1.38	0.16
Sex of the newborn—female	0.46	0.21
Sex of the newborn—male	3.22	0.21
Complications during pregnancy—presence	0.23	0.83
Complications during pregnancy—absence	1.61	0.83
Gestational background—multiparous	0.46	0.43
Gestational background—primiparous	2.07	0.43
Age of the mother—>36	1.61	0.0
Age of the mother—18–35	1.61	0.81
Age of the mother—14–18	1.84	0.01
Gestational age—post-term	0.46	0.02
Gestational age—premature	0.46	0.2
Gestational age—term	0.46	0.2

Note: Percentage (%): proportion of newborns affected by each characteristic relative to the total sample. *p*-Value: statistical significance level (*p* < 0.05 indicates a significant association).

## Data Availability

https://ucacueedu-my.sharepoint.com/:x:/g/personal/mvelezl_ucacue_edu_ec/EfhYqm0KkrlAvrkAbrT3snIBTpWkv3UsOCTsAkdOm7Dl8w?rtime=IsgxbLqs3EgAuthor (accessed on 1 May 2024).

## Abbreviations

The following abbreviations are used in this manuscript:

EPs	Epstein’s pearls
BNs	Bohn’s nodules
DLCs	Dental lamina cysts
CE	Congenital epulis
NT	Natal teeth
AG	Ankyloglossia
NNT	Neonatal teeth
